# ELF4 contributes to esophageal squamous cell carcinoma growth and metastasis by augmenting cancer stemness via FUT9: Role of ELF4 in esophageal squamous cell carcinoma

**DOI:** 10.3724/abbs.2023225

**Published:** 2023-09-07

**Authors:** Aiping Xu, Mingchuang Sun, Zhaoxing Li, Yuan Chu, Kang Fang, Yunwei Zhang, Jingjing Lian, Li Zhang, Tao Chen, Meidong Xu

**Affiliations:** 1 Endoscopy Center Zhongshan Hospital School of Medicine Fudan University Shanghai 200032 China; 2 Endoscopy Center Department of Gastroenterology Shanghai East Hospital School of Medicine Tongji University Shanghai 200120 China; 3 Department of Gastroenterology and Hepatology Jing’an District Centre Hospital Fudan University Shanghai 20032 China; 4 Department of Pathology Shanghai East Hospital School of Medicine Tongji University Shanghai 200120 China

**Keywords:** esophageal squamous cell carcinoma, transcription factor, ELF4, FUT9, stemness

## Abstract

Esophageal squamous cell carcinoma (ESCC) commonly has aggressive properties and a poor prognosis. Investigating the molecular mechanisms underlying the progression of ESCC is crucial for developing effective therapeutic strategies. Here, by performing transcriptome sequencing in ESCC and adjacent normal tissues, we find that E74-like transcription factor 4 (ELF4) is the main upregulated transcription factor in ESCC. The results of the immunohistochemistry show that ELF4 is overexpressed in ESCC tissues and is significantly correlated with cancer staging and prognosis. Furthermore, we demonstrate that ELF4 could promote cancer cell proliferation, migration, invasion, and stemness by *in vivo* assays. Through RNA-seq and ChIP assays, we find that the stemness-related gene fucosyltransferase 9 ( *FUT9*) is transcriptionally activated by ELF4. Meanwhile, ELF4 is verified to affect ESCC cancer stemness by regulating FUT9 expression. Overall, we first discover that the transcription factor ELF4 is overexpressed in ESCC and can promote ESCC progression by transcriptionally upregulating the stemness-related gene *FUT9*.

## Introduction

Esophageal cancer is an aggressive malignancy that causes over 400,000 deaths worldwide annually. Esophageal squamous cell carcinoma (ESCC) accounts for most esophageal cancers, especially in China [ 1, 2]. Although there are multiple therapeutic strategies, such as operative treatment, chemotherapy, and radiotherapy, poor prognosis and a low 5-year survival rate still exist [3]. Thus, more efforts should be made to deeply understand the biological behavior of esophageal squamous cell carcinoma and identify promising diagnostic markers and therapeutic targets by exploring the underlying molecular mechanisms.

The E26 transformation-specific (ETS) transcription factor family is an evolutionarily conserved and well-recognized group of transcription factors. To date, 28 members of the ETS family in humans and 27 in mice have been found. The unique structure of the ETS family is a DNA binding domain comprising 85 amino acids that can bind to the DNA sequence 5′-GGA(A/T)-3′ [ 4, 5]. Multiple ETS family members have been implicated in the pathogenesis and progression of various cancers. This can be attributed not only to their involvement in fundamental biological processes such as growth, proliferation, and differentiation but also to their regulatory roles in tumorigenesis, immunity, and basal cellular homeostasis. ELF4 is a member of the E74-like transcription factor (ELF) subfamily of the ETS family. Many previous studies have reported that ELF4 plays an important role in the immune response, osteogenesis, adipogenesis, cancer biology, and stem cell quiescence [6]. The aberrant interaction of ELF4 with the onco-fusion protein acute myeloid leukaemia (AML1)/ETO is an example of the involvement of ELF4 in cancer biology [7]. Additionally, increasing attention has been given to the association of ELF4 with solid tumors, such as primary human ovarian tumors and hepatocellular carcinoma [ 8, 9]. Although many studies have described ELF4 as an oncogenic protein, ELF4 can act as a tumor suppressor in some tumors, including lung adenocarcinoma and prostate tumors [ 10, 11]. ELF4, as a transcription factor, can regulate the transcription of a broad range of genes under specific conditions. Although the majority of studies have demonstrated that ELF4 is a potent transcriptional activator, there is also evidence of its functioning as a repressor. However, the specific role of ELF4 in esophageal squamous cell carcinoma (ESCC) remains unclear.

In the present study, we hypothesized that ELF4 might also play an important role in ESCC progression. Herein, we first explored the expression of ELF4 in ESCC samples and delineated its relationship with tumor progression and prognosis. We also investigated the function of ELF4 in affecting ESCC stemness, growth, and metastasis both *in vitro* and *in vivo*. The direct target gene and the underlying mechanism were also identified.

## Materials and Methods

### Data collection

In this study, we employed the transcriptome sequencing of ESCC and corresponding normal tissue from 6 patients in our center and delineated that ELF4 was a main upregulated transcription factor in ESCC tissues. In addition, through the UALCAN ( http://ualcan.path.uab.edu/) and Oncomine ( http://www.oncomine.com) databases, we verified the expression of ELF4 in ESCC from the TCGA and GEO platforms.

### Cell culture

Cell lines, including KYSE150, ECA109, and TE1, were purchased from the Institute of Biochemistry and Cell Biology of the Chinese Academy of Sciences (Shanghai, China). Cells were cultured in DMEM (Gibco, Carlsbad, USA) containing 10% fetal bovine serum (Gibco) under 5% CO _2_ at 37°C.

### Cell transfection

Cells were seeded in a 6-well plate and transfected with 100 pmol siRNA-NC (forward 5′-UUCUCCGAACGUGUCACGUTT-3′ and reverse 5′-ACGUGACACGUUCGGAGAATT-3′) or siRNA-ELF4 (#1, forward 5′-CCGCGGAAGUCUUACUCAATT-3′ and reverse 5′-UUGAGUAAGACUUCCGCGGTT-3′; #2, forward 5′-GGACCUGGUGGUCAUUGAATT-3′ and reverse 5′-UUCAAUGACCACCAGGUCCTT-3′; #3, forward 5′-CCUCCUAUGUUCAGGGUAUTT-3′ and reverse 5′-AUACCCUGAACAUAGGAGGTT-3′) or siRNA-FUT9 (#1, forward 5′-GGCACAAGAAACCUUAAGUTT-3′ and reverse 5′-ACUUAAGGUUUCUUGUGCCTT-3′; #2, forward 5′-CUCCAAUCCAUCUUAAUAATT-3′ and reverse 5′-UUAUUAAGAUGGAUUGGAGTT-3′; #3, forward 5′-GGAGAGUUAACAGUUAUUATT-3′ and reverse 5′-UAAUAACUGUUAACUCUCCTT-3′) (GenePharma, Shanghai, China) in each well for transient transfection. RT-qPCR and western blot analysis were utilized to evaluate the knockdown efficiency of 2 genes at 48 h after transfection. Concerning stable transfection, lentiviral vectors encoding a shRNA-NC (5′-TTCTCCGAACGTGTCACGT-3′) or shRNA-ELF4 (#1, 5′-CCCTGATTTACTGCATCTGTA-3′; #2, 5′-CTCTACTTTCAAGGACACCTT-3′; and #3, 5′-GCACTAAGATACTACTACCAA-3′) or lentiviral vectors inserted with FUT9 cDNA (#GOSL0334883) (Genechem, Shanghai, China) were used to transfect tumor cells according to the manufacturer’s instructions. In brief, when the cell density reached approximately 30% after being seeded in a 6-well plate, viral fluid mixed with a medium free of serum was added to the plate. After incubation for 24 h, the complete medium was replaced. The knockdown or overexpression efficiency was also detected by western blot analysis.

### Cell counting kit 8 assay

Cell counting kit 8 (CCK8) assay was used to explore the proliferation ability of cancer cells. As we performed previously [12], a total of 1×10 ^3^ cells were initially plated into each well of a 96-well plate, and 10 μL CCK8 reagent (Dojindo Laboratories, Kumamoto, Japan) was added to each well when the cells were cultured for 0, 24, 48, 72, and 96 h. After incubation for 2 h, the absorbance at 450 nm was measured with a microplate reader (Thermo Fisher, Waltham, USA).

### Cell colony formation assay

ECA109 and KYSE150 cells were initially plated in a 6-well plate (1000 cells/well) [13], and the complete medium was replaced every 2 days. After incubation for 2 weeks, the cells were fixed with 4% paraformaldehyde and stained with Giemsa. Under a 40× magnifying glass, the number of colonies (a single clone exceeded 50 cells) was manually counted.

### Tumor sphere-forming assay

ECA109 and KYSE150 cells were plated in superlow adherence dishes (Corning, New York, USA). The medium (DMEM-F12, B27, 20 ng/mL epidermal growth factor, and 20 ng/mL basic fibroblast growth factor) was replaced every 3 days. Spheres larger than 2 mm in diameter were counted, and images were taken under a light microscope (Olympus, Tokyo, Japan) at 50× magnification [14].

### Migration and invasion assay

Briefly, 5×10 ^4^ cells were resuspended in 200 μL DMEM free of serum and then added into each upper chamber of a Transwell device (8 μm pore size; Corning). Subsequently, 600 μL of complete medium was added into the lower chamber as the chemical attractant. After incubation for 24 h at 37°C, 4% paraformaldehyde was used to fix the cells on the lower surface of the noncoated membrane and then stained the migratory cells with Giemsa [15]. Under a light microscope (100×), images were taken from five representative fields of each membrane. By counting the migratory cells, the relative migration rate could be calculated. The invasion assay was performed similarly, with the only difference that before cells were added, a layer of gel formed from Matrigel matrix (356234; Corning) on the center of each Transwell insert was made first.

### Cell wound scratch assay

A cell wound scratch assay was performed as we did in our previous study [16]. The transfected ECA109 and KYSE150 cells were plated in a 6-well plate, and when approximately 80% of the surface was overspread with cells, a straight scratch was made in the monolayer with a 200-μL sterile pipette tip. After wash with PBS 2 times, the cells were cultured in DMEM containing 1% FBS. The wound was observed and photographed with a light microscope (100×) at 0, 24, and 48 h. Finally, the wound was calculated by ImageJ (NIH, Bethesda, USA), and the migration rate was calculated as follows: migration rate (%)=(scratch distance at 0 h‒scratch distance at 24 h or 48 h)/the scratch distance at 0 h×100%.

### RT-qPCR and RNA-seq

Trizol reagent (Invitrogen, Carlsbad, USA) was used to extract total RNA from ESCC cells following the manufacturer’s instructions. After RNA concentrations were measured with a Nanodrop2000 spectrophotometer (Thermo Fisher), a PrimeScript RT reagent kit (TaKaRa, Dalian, China) was used to synthesize complementary DNA. TaqMan real-time qPCR assays for *ELF4* and *FUT9* were performed, and *β-actin* was used as the internal reference. Sequences of primers are listed in Supplementary Table S1. All reactions were run in triplicate. The CT value calculation and the transcriptional level analysis were performed using the 2 ^–ΔΔCT^ method. RNA-seq of *ELF4*-knockdown cells was performed on the HiSeq2500 platform (Illumina, San Diego, USA).

### Western blot analysis

Tissues and cells were harvested and lysed in RIPA buffer supplemented with Protease Inhibitor Cocktail (Sigma-Aldrich, St Louis, USA). After the proteins were extracted, the concentrations were measured using a BCA kit (Beyotime Biotechnology, Shanghai, China), and proteins were separated by 8%‒12% SDS-PAGE and blotted onto PVDF membranes (Millipore, Billerica, USA). After being blocked with non-fat milk for 1 h, membranes were incubated with specific primary antibodies targeting ELF4 (sc-515363; Santa Cruz, Santa Cruz, USA), FUT9 (60230-1-lg; Proteintech, Chicago, USA), CD44 (ab264539; Abcam, Cambridge, UK), and CD133 (ab278053; Abcam) overnight at 4°C. After wash with TBST 3 times, the membranes were incubated with the corresponding secondary antibodies for 2 h at room temperature. Finally, the ECL luminescent solution was evenly spread onto the PVDF membrane, and the signals were detected. Antibodies against β-actin (ab8227; Abcam), GAPDH (Cat#2118; Cell Signaling Technology, Beverly, USA), and tubulin (Cat#2146; Cell Signaling Technology) were used as the loading controls for western blot analysis.

### Chromatin immunoprecipitation (ChIP) assay

The ChIP assay was performed as described previously [16]. Briefly, 1% formaldehyde was used to crosslink ECA109 cells for 10 min at 4°C. After cross-linking, SDS lysis buffer was used to extract cells. Sonication, centrifugation, and dilution were performed in ChIP dilution buffer. Chromatin from crosslinked ECA109 cells was incubated overnight with anti-ELF4 or normal rabbit IgG, followed by incubation with protein G-Sepharose saturated with salmon sperm DNA. The amount of precipitated DNA was measured by RT-qPCR. The enrichment value was calculated based on the relative amount of input and the IgG ratio. The primers covering the ELF4 binding site of the *FUT9* gene promoter region are shown in Supplementary Table S1.

### Dual luciferase reporter assay

ESCC cells were seeded into a 12-well plate. When the surface of the well was covered over 80% by cells, the reporter plasmid comprising the wild-type FUT9 promoter or mutant FUT9 promoter at the binding site was transfected into cells using Lipofectamine 3000 (Invitrogen) according to the manufacturer’s instructions. Signal normalization was performed by *Renilla* luciferase transfection. The Dual Luciferase Assay System kit (Promega, Madison, USA) was used to measure the luciferase activity at 24 h after transfection.

### Flow cytometric analysis

For CD44 ^+^ cell analysis, flow cytometry was performed. Based on the standard protocol [17], cells were incubated with anti-CD44 (ab264539; Abcam) for 2 h at 4°C. Subsequently, the cells were incubated with the secondary antibody anti-mouse Alexa 488 (ab150113; Abcam) for 30 min at room temperature in the dark and detected with a flow cytometer (CytoFLEX; Beckman, Pasadena, USA).

### Immunohistochemistry (IHC) assay

ESCC and adjacent healthy esophageal mucosa tissues of 79 patients were obtained. Written informed consent was obtained from all enrolled patients. The operation of this study was approved by the Ethics Committee of Shanghai East Hospital, Tongji University (2019-072). The tissue samples were fixed, embedded, and sectioned (4 μm thickness) [12]. An antigen retrieval solution and a blocking solution were added to the sections after dewaxing. Primary antibodies including anti-ELF4 (sc-515363; Santa Cruz), anti-FUT9 (60230-1-lg; Proteintech), anti-CD44 (ab264539; Abcam), and anti-CD133 (ab278053; Abcam) were applied to the slides and incubated overnight at 4°C. After counterstaining with light hematoxylin, the slides were dehydrated, sealed with cover glass, and examined under a light microscope. The results were scored by two pathologists who were blinded to patient information.

### Animal studies

The Animal Care and Use Committee of Tongji University approved this *in vivo* study (TJBB05222101). All mice received humane care according to the criteria outlined in the Guide for the Care and Use of Laboratory Animals published by the National Institutes of Health (Bethesda, USA). Twelve female BALB/c nude mice (6 weeks old; GemPharmatech, Nanjing, China) were used in this *in vivo* study. The animals were randomly divided into control and treatment groups, and each group comprised 6 mice. First, stable transfection was applied to ECA109 cells. Cells were collected and resuspended for subcutaneous injection into the right flanks of each mouse (5×10 ^6^ cells/mouse) [12]. After 6 weeks, all mice were euthanized according to the AVMA Guidelines for the Euthanasia of Animals. In brief, a 3-fold dose of barbiturates was intraperitoneally injected into the mice. After the mice were euthanized, the subcutaneous tumors were removed, photographed and weighed. The tumor volume was exhibited by the maximum cross-sectional area of the tumor. The tumor volume was measured by using length and width with a vernier caliper and calculated using the equation: tumor volume=(length×width ^2^)/2. After the tumors were weighed, each tumor was cut into two parts on average. One part of each tumor was fixed with 10% phosphate-buffered formaldehyde and embedded in paraffin for IHC analysis. The rest was subject to western blot analysis.

### Statistical analysis

Data were analyzed with SPSS 19.0 (IBM, Armonk, USA). Comparisons between groups were performed by Student’s *t* test, the chi-square test, or Fisher’s exact test. Kaplan-Meier methods and log-rank tests were used to analyze survival data. A *P* value<0.05 was considered statistically significant.

## Results

### ELF4 expression is upregulated in esophageal squamous cell carcinoma

**Figure 1:**
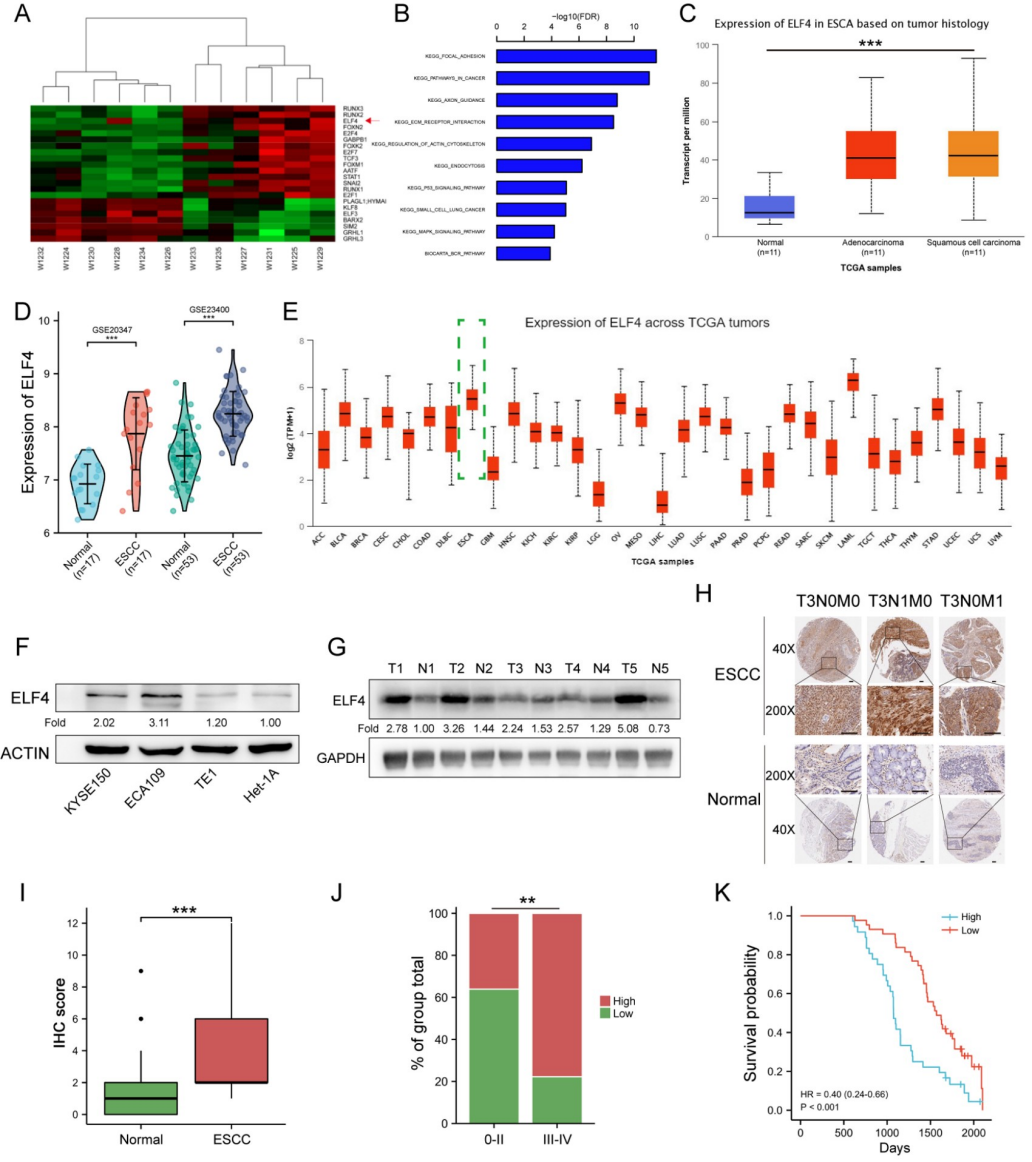
Figure 1 ELF4 is overexpressed and correlated with tumor progression and prognosis in ESCC (A) The sequencing data indicated that ELF4 was a significantly upregulated transcription factor in our ESCC samples. (B) KEGG analysis showed that the upregulated genes were mostly involved in 10 pathways. The expression level of ELF4 mRNA was elevated in ESCC tissues by analyzing the TCGA (C) and GEO (D) databases. (E) Pan-cancer analysis of ELF4 expression levels showed that esophageal carcinoma ranked the second. (F,G) Western blot analysis showed that ELF4 was elevated in ESCC cell lines and our randomly selected ESCC tissues. (H,I) Immunohistochemistry analysis showed that the expression level of ELF4 was higher in our ESCC tissues. (J) The protein level of ELF4 was higher in stage III‒IV ESCC. (K) Kaplan-Meier analysis indicated that a high expression level of ELF4 was associated with reduced overall survival in our clinical tissue samples. **P<0.01, and ***P<0.001. Scale bar: 100 μm.

To identify the potential pathological drivers of ESCC, we performed transcriptome sequencing. We screened the global expression profiling of protein-coding transcripts in 6 ESCC tissues and matched normal esophageal mucosa tissues. Using defined criteria of FC ≥ 2 and *P*<0.05, we identified 22 transcription factors (15 upregulated and 7 downregulated genes) that were differentially expressed between the tumor and normal tissues ( Figure 1A). KEGG analysis indicated that the upregulated genes were involved in 10 pathways ( Figure 1B). Based on altered transcription factor expression levels and KEGG analysis, ELF4 was selected for further study. Meanwhile, we analyzed the online data from TCGA and GEO datasets and found that in both databases, the expression of ELF4 was significantly upregulated in ESCC ( Figure 1C,D). Additionally, pan-cancer analysis of the expression of ELF4 in TCGA showed that the expression of ELF4 in esophageal carcinoma ranked second and was only lower than that in acute myeloid leukemia tumor ( Figure 1E). We applied western blot analysis to investigate the expression of ELF4 protein in ESCC cell lines and the normal epithelial cell line of the esophagus. We found that ELF4 was significantly upregulated in ESCC cell lines ( Figure 1F). In addition, we randomly selected 5 pairs of samples from ESCC patients and explored the expression of ELF4. Western blot analysis results showed that the expression of ELF4 protein in ESCC tissues was obviously higher than that in paired normal esophageal mucosa tissues ( Figure 1G). This result was further confirmed by immunohistochemistry analysis in ESCC tissues ( Figure 1H,I).

### Elevated expression of ELF4 protein is associated with poor prognosis of ESCCs

After analyzing the correlation between clinicopathologic parameters and the expression level of ELF4 in ESCC tissues, we observed that the expression level of ELF4 protein in stage III-IV tumors was higher than that in stage 0‒II tumors ( Figure 1J). Additionally, Kaplan-Meier analysis in our study indicated that patients with low expression of ELF4 protein had significantly better overall survival ( Figure 1K).

### ELF4 promotes ESCC cell proliferation, migration, invasion, and stemness

**Figure 2:**
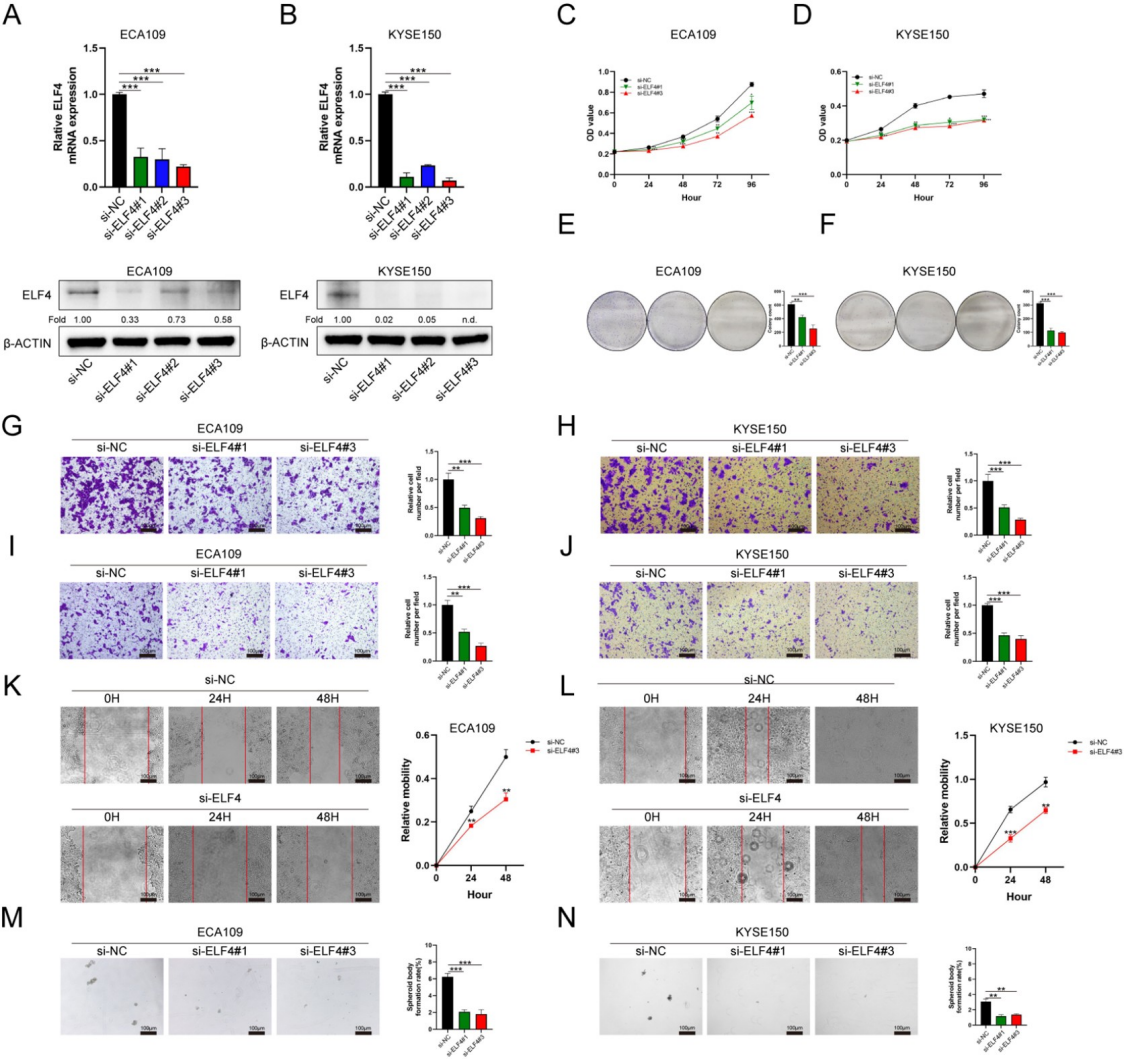
Figure 2 ELF4 affects the proliferation, migration, invasion, and stemness of ESCC cells (A,B) RT-qPCR and western blot analysis indicated that ELF4 was significantly inhibited by siELF4 in ECA109 and KYSE150 cells. (C,D) CCK8 assay indicated that ELF4 knockdown inhibited the proliferation ability of ECA109 and KYSE150 cells. (E,F) Cell colony formation assays further validated the proliferation inhibition. (G,H) ELF4 knockdown significantly blunted the migration ability of ECA109 and KYSE150 cells in the migration assay. (I,J) ELF4 knockdown attenuated the invasion ability of ECA109 and KYSE150 cells in the invasion assay. (K,L) Wound scratch assay further validated the migration inhibition. (M,N) Tumor sphere-forming assay indicated that ELF4 knockdown reduced cancer stem-like properties. **P<0.01, and ***P<0.001. Scale bar: 100 μm.

To further investigate how ELF4 affects the abilities of ESCC cells, *in vitro* assays, including CCK8, cell colony, migration, invasion, wound scratch, and tumor sphere-forming assays, were performed. The knockdown efficiency of specific siRNA targeting ELF4 was confirmed by RT-qPCR and western blot analysis ( Figure 2A,B). The results of CCK8 and cell colony assays indicated that the proliferation of ECA109 and KYSE150 cells was significantly inhibited when *ELF4* was knocked down ( Figure 2C–F). In addition, from the results of migration, invasion, and wound scratch assays, we found that the migration and invasion abilities of ESCC cells were also obviously attenuated when *ELF4* was knocked down ( Figure 2G‒L). The results of tumor sphere-forming assays showed that *ELF4* knockdown could also reduce the cancer stem-like properties of ESCC cells ( Figure 2M,N).

### ELF4 transcriptionally activates *FUT9* and further affects the expressions of cancer stemness-related markers

**Figure 3:**
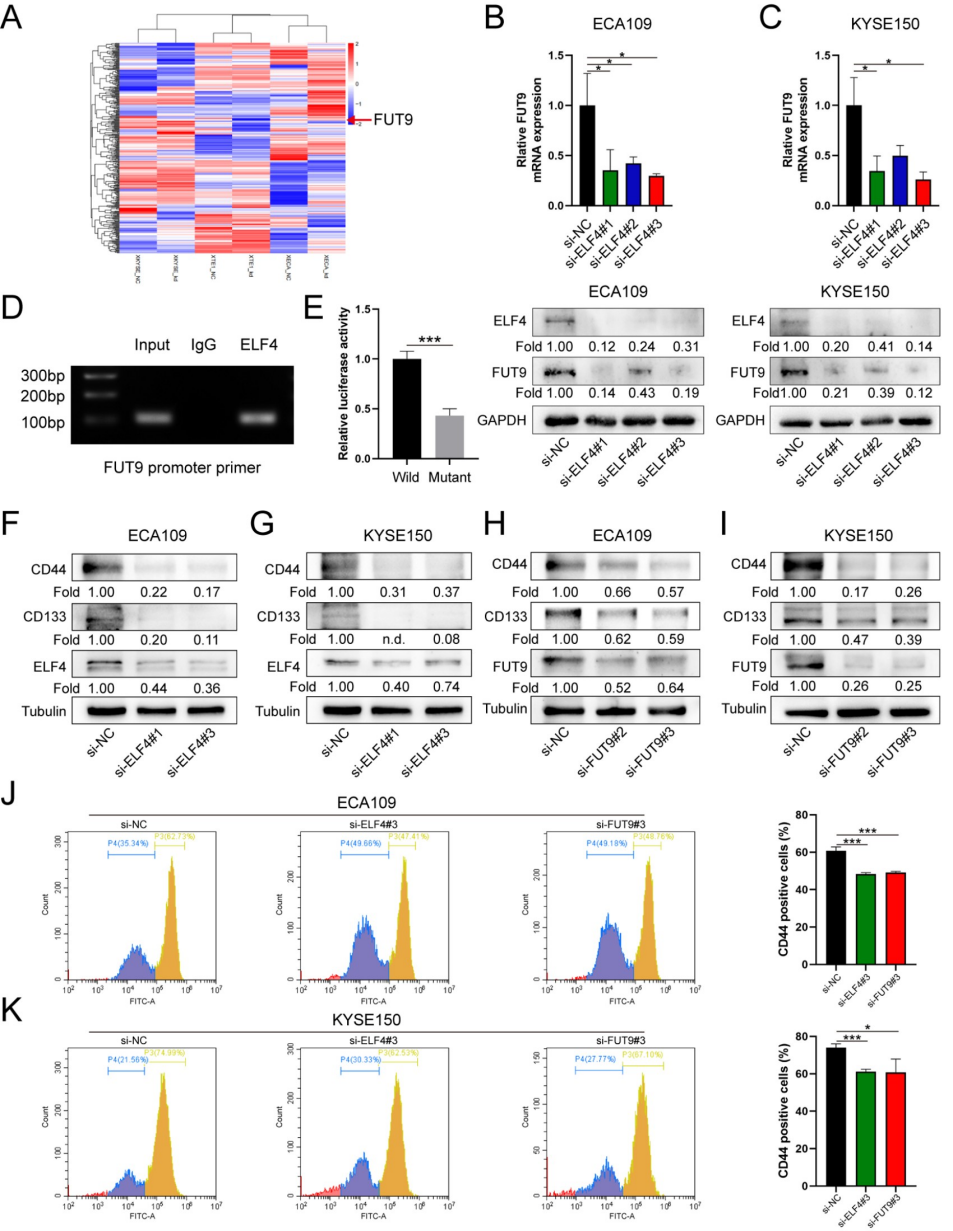
Figure 3 ELF4 augments the cancer stem-like properties of ESCC cells by transcriptionally upregulating FUT9 expression (A) Clustering analysis of RNA-seq revealed significantly upregulated and downregulated genes in the KYSE150, ECA109, and TE1 siELF4 groups normalized to the control groups. (B,C) RT-qPCR and western blot analysis indicated that the knockdown of ELF4 could attenuate FUT9 expression in ECA109 and KYSE150 cells. (D) ChIP assay showed that a positive signal was obtained through primers targeting FUT9. (E) Luciferase reporter gene assays revealed that mutations at the predicted binding site of FUT9 could significantly decrease luciferase activity in ECA109 cells. (F,G) Western blot analysis indicated that ELF4 knockdown could attenuate the expression of cancer stemness markers. (H,I) Western blot analysis indicated that FUT9 knockdown attenuated the expressions of cancer stemness markers. (J,K) Flow cytometric analysis revealed that the proportion of CD44+ cells was reduced when ELF4 or FUT9 was knocked down. *P<0.05, and ***P<0.001.

To explore the underlying molecular mechanism of ELF4 in promoting ESCC progression, we performed an RNA-seq analysis. We explored the alteration of protein-coding transcript levels in TE1, KYSE150, and ECA109 cells treated with or without siELF4. With *P*<0.05 and FC≥4 as cut-off values, 38 downregulated genes in cells transfected with siELF4 were detected ( Figure 3A). Among the 38 genes, we focused on the gene *FUT9* because of its close relationship with cancer stemness reported in other cancers. We performed RT-qPCR and western blot analysis and found that the expression of FUT9 was significantly downregulated in ECA109 and KYSE150 cells transfected with siELF4 ( Figure 3B,C). Considering that ELF4 is a transcription factor, we hypothesized that ELF4 might function by regulating the transcription of the *FUT9* gene. Consequently, the ChIP assay verified the binding site of ELF4 in *FUT9* ( Figure 3D). The results of luciferase assays also demonstrated that ELF4 could transcriptionally activate FUT9 ( Figure 3E). Furthermore, western blot analysis results showed that the expressions of the cancer stemness markers CD44 and CD133 were significantly decreased when *ELF4* or *FUT9* was knocked down ( Figure 3F‒I). By flow cytometric analysis, we also found that the proportion of CD44 ^+^ (Gate: P3) cells was reduced when *ELF4* or *FUT9* was knocked down ( Figure 3J,K).

### ELF4 and FUT9 are positively correlated with the expressions of the cancer stemness markers CD44 and CD133 in clinical samples

**Figure 4:**
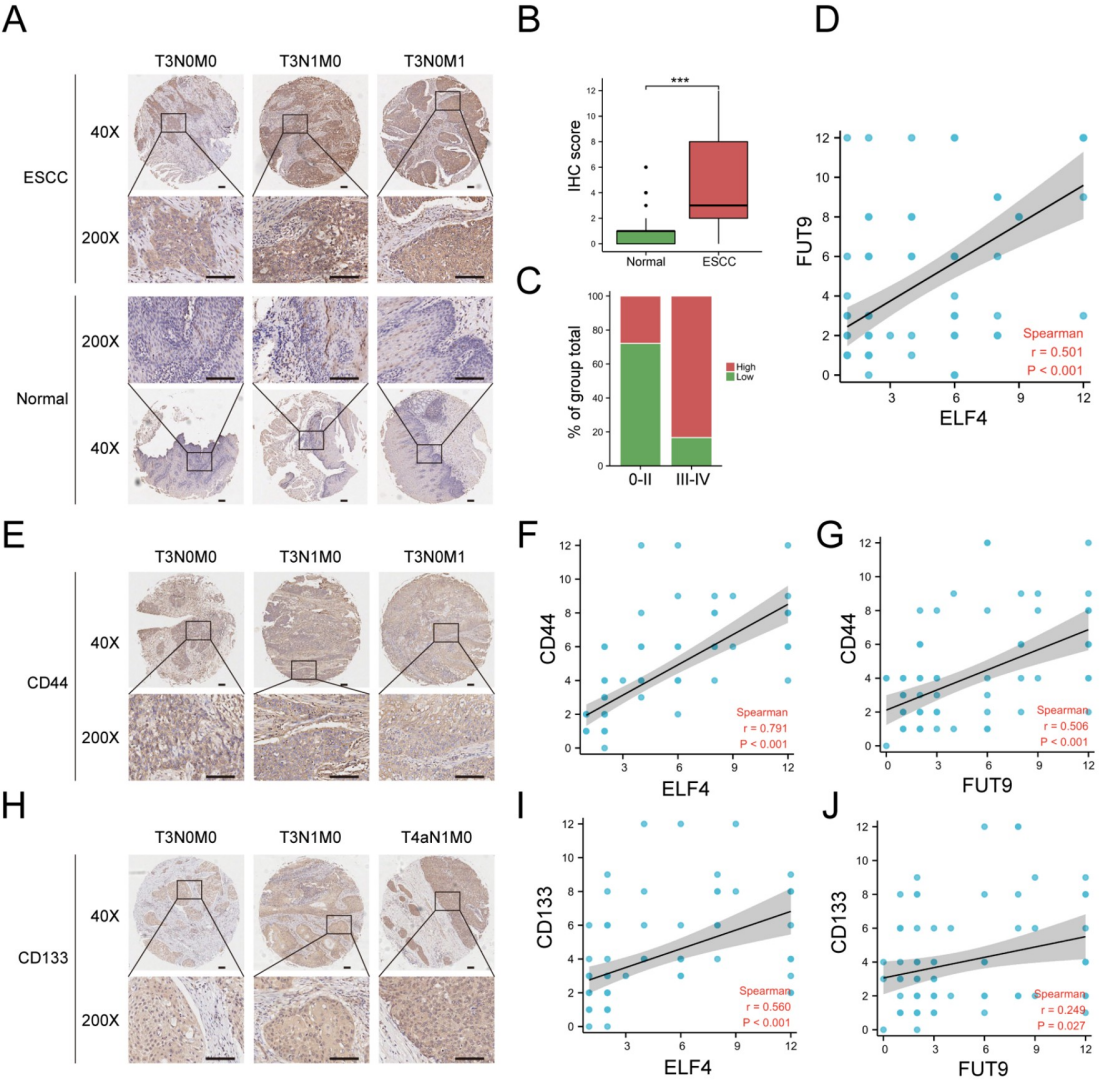
Figure 4 ELF4 expression is positively correlated with FUT9 expression and cancer stemness marker expressions in ESCC tissues (A,B) Immunohistochemistry indicated that FUT9 was overexpressed in our ESCC tissues. (C) The protein level of FUT9 was higher in stage III‒IV ESCC. (D) Correlation analysis revealed that ELF4 expression was positively correlated with FUT9 expression. (E‒J) Immunohistochemistry analysis indicated that ELF4 and FUT9 expression levels were positively correlated with the cancer stemness markers CD44 and CD133, respectively. ***P<0.001. Scale bar: 100 μm.

By immunohistochemistry analysis, we found that the expression of FUT9 was upregulated in ESCC tissues and related to tumor progression ( Figure 4A–C). Correlation analysis further validated that the expression of ELF4 in our ESCC tissues was positively correlated with the expression of FUT9 ( Figure 4D). Additionally, the expression levels of both ELF4 and FUT9 were significantly positively correlated with the expressions of the cancer stemness markers CD44 and CD133 ( Figure 4E–J).

### FUT9 induces ESCC cell proliferation, migration, invasion, and stemness

**Figure 5:**
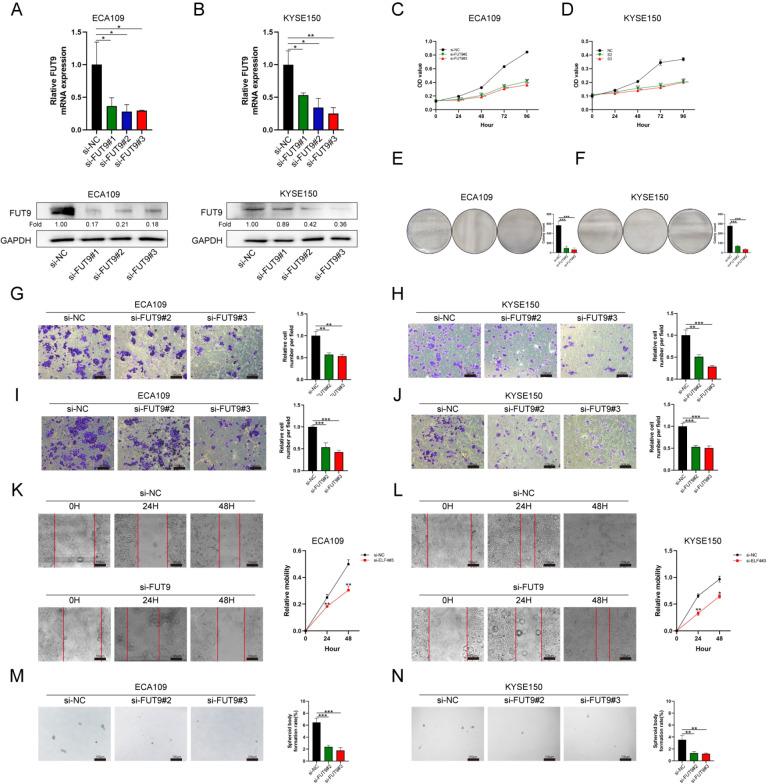
Figure 5 FUT9 affects the proliferation, migration, invasion, and stemness of ESCC cells (A,B) RT-qPCR and western blot analysis revealed that FUT9 expression was significantly suppressed by siFUT9 in ECA109 and KYSE150 cells. (C,D) CCK8 assay indicated that FUT9 knockdown inhibited the proliferation ability of ECA109 and KYSE150 cells. (E,F) Cell colony formation assays further validated the proliferation inhibition. (G,H) FUT9 knockdown significantly blunted the migration ability of ECA109 and KYSE150 cells in the migration assay. (I,J) FUT9 knockdown obviously inhibited the invasion of ECA109 and KYSE150 cells in the invasion assay. (K,L) Wound scratch assay further validated the migration inhibition. (M,N) Tumor sphere-forming assay indicated that FUT9 knockdown reduced cancer stem-like properties. *P<0.05, **P<0.01, and ***P<0.001. Scale bar: 100 μm.

To explore the effects of FUT9 on ECA109 and KYSE150 cells, CCK8, cell colony, migration, invasion, wound scratch, and tumor sphere-forming assays were carried out. The results of RT-qPCR and western blot analysis indicated that the expression of FUT9 was significantly downregulated after siFUT9 transfection ( Figure 5A,B). The results of CCK8 and cell colony assays revealed that the cell proliferation ability in these two cell lines was suppressed when *FUT9* was knocked down ( Figure 5C‒F). In addition, the migration and invasion abilities were also significantly suppressed when *FUT9* was knocked down, as indicated by migration, invasion, and wound scratch assays ( Figure 5G‒L). The tumor sphere-forming assays showed that cancer stem-like properties were reduced when *FUT9* was knocked down ( Figure 5M,N).

### Ectopically expressed FUT9 rescues the inhibitory effects of *ELF4* knockdown on the proliferation, migration, invasion and stemness of ESCC cells

We performed rescue experiments to further verify that ELF4 promotes ESCC progression by transcriptionally activating FUT9. First, western blot analysis results showed that FUT9 was overexpressed after lentiviral vectors carrying FUT9 cDNA were transfected into cells ( Supplementary Figure S1A,B). We then transfected FUT9 cDNA-inserted lentiviral vectors into *ELF4*-silenced ESCC cells and performed *in vitro* assays. The results of the cell colony assay showed that the inhibitory effects of *ELF4* knockdown on cell proliferation in these two cell lines could be rescued by ectopically expressed FUT9 ( Supplementary Figure S1C,D). In addition, the suppressive effects of *ELF4* knockdown on cell migration and invasion were also rescued, as revealed by migration and invasion assays ( Supplementary Figure S1E‒H). After lentiviral vectors carrying FUT9 cDNA were transfected, the inhibitory effects of *ELF4* knockdown on cancer stemness could also be significantly rescued ( Supplementary Figure S1I,J).

### Effect of ELF4 on tumorigenesis *in vivo*

**Figure 6:**
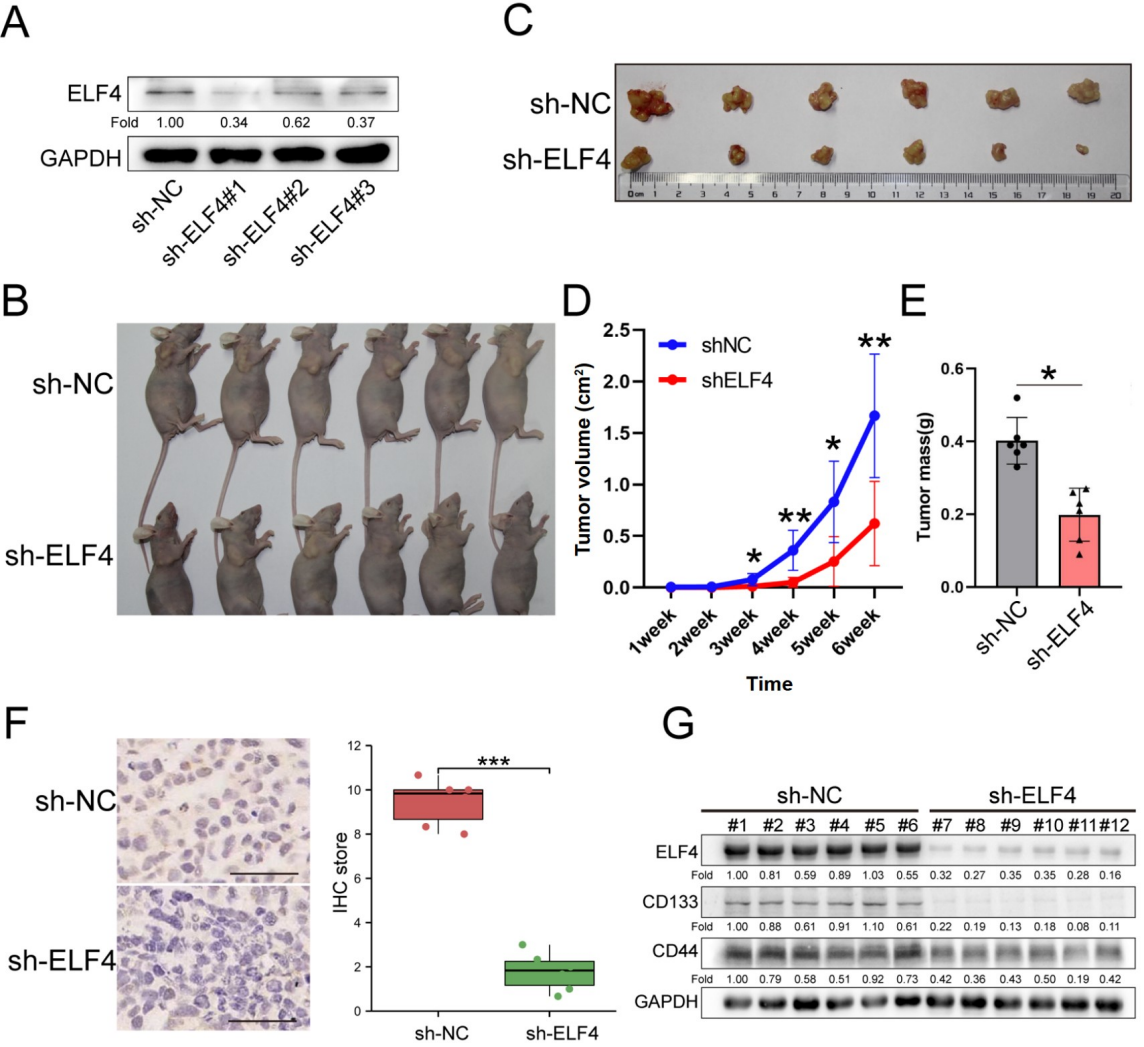
Figure 6 ELF4 affects tumor growth *in vivo* (A) Western blot analysis revealed that ELF4 was significantly inhibited by Lv-shELF4 in ECA109 cells. (B,C) ELF4 knockdown inhibited subcutaneous tumorigenesis in nude mice after ECA109-derived cell injection. (D) The growth curves of the tumors indicated that ELF4 knockdown inhibited subcutaneous tumor growth. (E) The weight of tumors removed from the nude mouse model indicated that tumors in the sh-ELF4 group weighed approximately 2.03 times less than the tumors observed in the control group. (F) Immunohistochemistry further verified the efficacy of ELF4 knockdown. (G) Western blot analysis validated that ELF4 was significantly knocked down in the sh-ELF4 group and that the cancer stem markers CD44 and CD133 were downregulated accordingly. *P<0.05, **P<0.01, ***P<0.001. Scale bar: 50 μm.

We utilized a mouse model to study the effect of ELF4 on ESCC tumorigenesis. The high knockdown efficiency of lentiviral vectors encoding shRNA targeting ELF4 was first validated ( Figure 6A). In the subcutaneous injection nude mouse model, the size of tumors in the sh-ELF4 group was obviously smaller, and the weight was approximately 2.03 times less than that in the control group ( Figure 6B‒E). Immunohistochemistry analysis confirmed that the expression of ELF4 was downregulated in the sh-ELF4 group ( Figure 6F). Furthermore, the results of the western blot analysis in these tumors validated that the expression levels of the cancer stemness markers CD44 and CD133 were decreased in the sh-ELF4 group ( Figure 6G).

## Discussion

ELF4, one of the ETS transcription factors, has been implicated in the pathogenesis and progression of various types of cancers. However, its specific role in ESCC remains unknown. In our study, we conducted transcriptome sequencing on ESCC tissues and matched normal esophageal mucosa tissues. Our analysis revealed that ELF4 was significantly upregulated in ESCC, suggesting its potential involvement in the disease. Cancer tissue consists of heterogeneous cell types, and each subpopulation has plasticity to turn into the others. Cancer stem cells (CSCs) are a subpopulation that possesses therapeutic resistance and tumor reconstruction capability and are responsible for cancer progression [ 18‒ 20]. Considering that ELF4 was reported to affect cancer stemness in many cancers [ 21, 22], we performed relevant *in vitro* and *in vivo* assays. From the results of these assays, ELF4 was validated to augment ESCC proliferation, migration, invasion, and cancer stem-like properties. Thus, in this study, we found that ELF4 might promote the progression of ESCC by increasing ESCC cancer stem-like properties.

Considering that ELF4 is a transcription factor, similar to its function in gliomas [22], there might be some target genes that ELF4 transcriptionally regulates in ESCC. Through RNA-seq, FUT9, a glycosyltransferase reported to affect cancer stem-like properties in many cancers, attracted our attention. Glycosylation, the fundamental posttranslational modification of proteins and lipids, is required for various biological and cellular functions [23]. As a common posttranslational modification in all organisms, fucosylation is based on the enzymatic activities of glycosyltransferases and glycosidases [24] and is increased in several cancers [25]. In this study, our immunohistochemistry analysis of the samples from ESCC patients verified higher FUT9 expression in ESCC tissues than that in normal tissues. By *in vitro* assays, FUT9 was validated to promote ESCC cell proliferation, migration, invasion, and stemness. Furthermore, ectopically expressed FUT9 dramatically rescued the inhibitory effects of *ELF4* knockdown on the proliferation, migration, invasion, and cancer stemness of ESCC cells. There was a strong possibility that ELF4 augmented ESCC cancer stem-like properties and further accelerated ESCC progression by transcriptionally regulating the expression of FUT9. We also demonstrated that inhibition of ELF4 significantly reduced the cancer-promoting capacity of the ELF4-FUT9 axis *in vitro* and its oncogenicity *in vivo*.

In addition to the observed positive correlation between ELF4 or FUT9 and the cancer stemness-related markers CD44 and CD133, our immunohistochemistry results revealed a significant positive correlation between the expressions of ELF4 and FUT9. Furthermore, when *ELF4* was knocked down, we observed a clear downregulation of FUT9 expression, as confirmed by RT-qPCR and western blot analysis. Notably, our ChIP and dual luciferase reporter assays provided evidence that FUT9 can be transcriptionally activated by ELF4, a finding that is novel in the context of ESCC. Moreover, due to early metastasis, most ESCC patients lose the best time for new therapeutic methods, such as endoscopic submucosal dissection (ESD). In this study, the relationship between the ELF4/FUT9 axis and the progression of ESCC was first elucidated, and ELF4 might be a novel promising diagnostic mrker and therapeutic target.

In summary, our study demonstrated that ELF4 is a critical transcription factor that affects cancer stemness in ESCC. In addition, the expression level of ELF4 is significantly related to ESCC progression and prognosis. ELF4 can augment cancer stemness and further promote tumor growth and metastasis in ESCC.

## Supporting information

Supplementary

## Supplementary Data

Supplementary data is available at *Acta Biochimica et Biophysica Sinica* online.
