# Supplementary material for: ELF4 contributes to esophageal squamous cell carcinoma growth and metastasis by augmenting cancer stemness via FUT9: Role of ELF4 in esophageal squamous cell carcinoma
Source: Acta Biochim Biophys Sin (Shanghai). 2023 Sep 7;56(1):129–39. doi: 10.3724/abbs.2023225 (PMC10875363; doi:10.3724/abbs.2023225)
Supplement: Supplementary [file Supplementary.pdf]

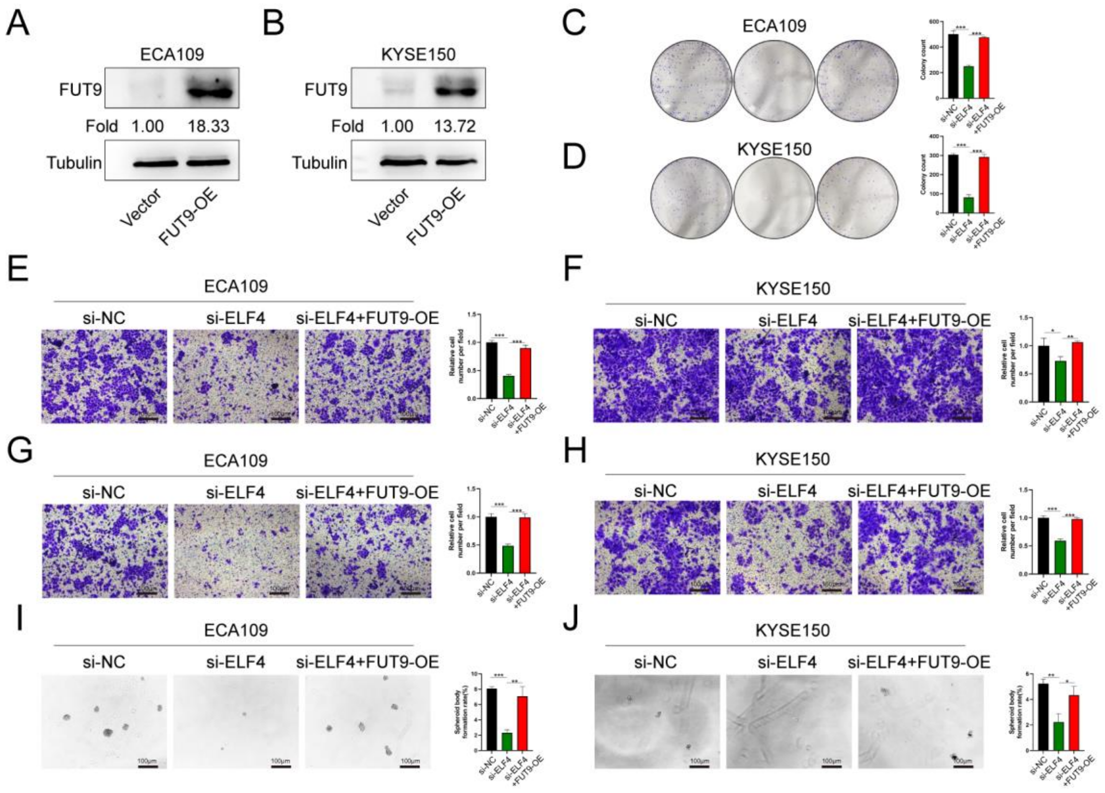

Supplementary Figure S1. Overexpression of FUT9 rescues the inhibitory effects of *ELF4* knocking down on ESCC cells *in vitro* (A,B) Western blot analysis revealed that FUT9 was significantly overexpressed in ECA109 and KYSE150 cells after infection with lentivirus vectors inserted with FUT9 cDNA. (C,D) Cell clone assay showed that the inhibitory effects of ELF4 downregulation on proliferation in ECA109 and KYSE150 cells were rescued by FUT9 overexpression. (E,F) FUT9 overexpression significantly rescued the migration ability of ECA109 and KYSE150 cells when *ELF4* was knocked down. (G,H) FUT9 overexpression significantly rescued the invasion ability of ECA109 and KYSE150 cells when *ELF4* was knocked down. (I,J) Tumor sphere-forming assay indicated the inhibitory effects of ELF4 downregulation on cancer stem-like properties were rescued in ESCC cells when FUT9 was overexpressed.

Supplementary Table S1. Sequences of primers using for real-time qPCR and CHIP assays

**Gene**	**Primer sequence**	
ELF4	Forward	5′-TTCATCAGGACTTCTGGCACTA-3′
	Reverse	5′-CACCATACCCTGAACATAGGAG-3′
FUT9	forward	5′-CCAGTGAGCTAGCAAAGTATCT-3′
	reverse	5′-TTGATGCCTTTTCACATGATCG-3′
FUT9 (for CHIP)	forward	5′-CCAGCCTGAGCCACGAGA-3′
	reverse	5′-GCAGGAGCCCAGCAGAGG-3′
